# Management of hypertension in chronic kidney disease

**DOI:** 10.1016/j.clinme.2026.100583

**Published:** 2026-04-24

**Authors:** Tim Doulton, Pauline A. Swift, Swapnil Hiremath

**Affiliations:** a Kent Kidney Care Centre, East Kent Hospitals University NHS Foundation Trust, Ethelbert Road, Canterbury, Kent CT1 3NG, UK; b South West Thames Renal and Transplantation Unit, Epsom & St Helier University Hospitals NHS Trust, Wrythe Lane, Carshalton, Surrey SM5 1AA, UK; c Department of Medicine, University of Ottawa, 1967 Riverside Drive, Ottawa, Ontario K1H7W9, Canada

**Keywords:** Blood pressure, Salt, Renin-angiotensin system inhibitors, Sodium-glucose co-transporter inhibitors, Non-steroidal mineralocorticoid receptor antagonists

## Abstract

Hypertension is a major risk factor for cardiovascular (CV) disease and progression in chronic kidney disease (CKD). Blood pressure (BP) measurement should be standardised and supplemented by ambulatory and home BP monitoring. BP goals are generally <140/90 and <130/80 mmHg for those at higher risk of CV disease or CKD progression. Lifestyle modification includes restricting salt intake to <5 g/day. Most people with hypertension and CKD, especially those with a urine albumin:creatinine ratio ≥3 mg/mmol, should be treated with a renin–angiotensin system inhibitor to which, in selected individuals, a sodium–glucose co-transporter inhibitors type 2 and non-steroidal mineralocorticoid receptor antagonist may be added, followed by additional antihypertensives to achieve goal BP. Strategies to manage hyperkalaemia (eg low potassium dietary advice and oral potassium binders) should be employed to avoid stopping prognostically beneficial medications. A range of emerging therapeutics, including aldosterone synthase and endothelin antagonists, and renal denervation are briefly discussed.

## Highlights

• Guidelines recommend goal BP <130/80 mmHg if high risk of CVD or CKD progression.
• First-line medication is generally a renin–angiotensin system inhibitor (RASi).
• Thiazide-like diuretics effectively reduce BP down to an eGFR of 15 mL/min/1.73 m^2^.
• Continue RASi even when eGFR is very low, unless refractory hyperkalaemia occurs.
• Use low potassium diet and oral potassium binders to avoid stopping RASi.

## Introduction

Raised blood pressure (BP) is highly prevalent among people with chronic kidney disease (CKD) and is a major risk factor for cardiovascular (CV) disease and CKD progression, particularly in those with higher levels of albuminuria. There are significant challenges when managing hypertension in CKD, most especially in those with low eGFR, frailty and/or multiple coexistent comorbidities, where polypharmacy is often necessary. Pharmacological interventions can impact eGFR and serum potassium, leading to therapeutic interruptions with subsequent undermanagement of kidney and CVD risk. This review aims to equip the non-specialist clinician with an overview of current best practice and provide a brief synopsis of emerging therapeutics in this field.

## Blood pressure measurement

BP measurement in clinical trials has always been rigorously conducted with proper resting, multiple measurements and trained observers. In the last two decades, automated BP measurement has allowed this to be further standardised – and the widespread availability of these devices makes it possible to translate trial-level measurement practices to everyday clinical practice. The difference between these ‘standardised’ measurements and a casual single BP is very large: the mean difference is about 7–12 mmHg in systolic BP, but the spread can be much wider (from +17 to −33 mmHg).^1^ These BP measurements do not need to be unattended,^1^, ^2^ and clinic workflow does need to be designed to allow smoother implementation of standardised BP measurements, such as measurement in the waiting room, or measurement in the clinic room while charting. Standardised BP lowering is especially important when aiming for intensive BP lowering (see below under BP targets). Ambulatory BP measurement is quite useful to augment BP management in CKD beyond ruling out white coat hypertension, since masked hypertension is much more common in CKD, as is nocturnal hypertension, both of which have prognostic implications.^3^ Home BP measurement is an alternative to ambulatory BP, with data supporting better outcomes and patient empowerment from the general population.^4^

## Blood pressure targets

BP targets differ somewhat among different professional societies although these have, over the years, drifted down towards 130/80 mmHg, as trial evidence has accumulated demonstrating lower CV event rates with intensive BP lowering^5^ (see Table 1). The Kidney Disease Improving Global Outcomes (KDIGO) guidelines are at the lower end, with a systolic BP target of <120 mmHg and requiring standardised BP measurement. However, even the other guidelines take a risk-based approach, with lower targets often considered, or suggested in the text, for higher-risk patients (notably with diabetes and/or proteinuria). Rather than consider the differences in societal guidance as a buffet of choice, this risk-based approach of targeting <140/90 mmHg in everyone, and <130/80 mmHg in patients at higher CV risk, keeping in mind individual patient goals and adverse event profile, is a sensible option. Clinical trials do exclude patients with standing systolic BP <110 mmHg, those at high risk of falls and those living in nursing homes, all conditions and scenarios that are common in people with CKD and in whom intensive BP targets should be avoided, irrespective of CV risk. Those with advanced CKD (eGFR <20 mL/min/1.73 m^2^) have been excluded from all large CV outcome trials. In these patients, the concern of worsening kidney function with intensive BP lowering must be balanced against the CV benefit and is accompanied by higher risk of adverse effects (eg hyponatraemia, hyperkalaemia) with pharmacotherapy. A small pilot trial (with achieved clinic BP of 125 mmHg versus 138 mmHg) reported no safety signals, and we await larger trials to guide.^6^

**Table 1:** Comparison of recommendations from selected international learned societies for BP targets in CKD.

	*KDIGO* *(2020)*	*ESH (2024)*	*NICE; UKKA (2021)*	*ESC (2024)*	*ACC/AHA* *(2025)*	*HT Canada* *(2025)*
*CKD* *No DM*	SBP <120	SBP 120–139 DBP 70–79	SBP 120–139 DBP 70–79	SBP 120–129 DBP 70–79	SBP <130	SBP <130
*CKD* *DM*	SBP <120	SBP 120–139 DBP 70–79	SBP 120–29^a^ DBP <80	SBP 120–129 DBP 70–79	SBP <130	SBP <130
*Advanced CKD* *(GFR <30)*	SBP <120	No separate recommendation	No separate recommendation	Individualised targets	No separate recommendation	No separate recommendation
*Renal transplant*	130/80	No separate recommendation	No separate recommendation	Individualised targets	Insufficient evidence	No separate recommendation

KDIGO, Kidney Disease, Improving Global Outcomes^7^; ESH, European Society of Hypertension^8^; NICE, National Institute for Health and Care Excellence^9^; UKKA, United Kingdom Kidney Association^10^; ESC, European Society of Cardiology^11^; ACC/AHA, American Society of Cardiology/American Heart Association^12^; HT Canada, Hypertension Canada^13^; CKD, Chronic Kidney Disease; DM, Diabetes mellitus; GFR, glomerular filtration rate, in mL/min/1.73 m^2^; SBP, systolic BP; DBP, diastolic BP.

a This target is also recommended for those with proteinuria, urine protein:creatinine ratio >70 mg/mmol.

## Lifestyle modifications

Dietary salt reduction is widely advocated in hypertension guidelines, including in those for people living with CKD. Reducing salt intake from 10 to 4 g per day in CKD stages 3 and 4 lowers office BP by 10/4 mmHg and decreases proteinuria by 33%.^14^ A subsequent meta-analysis of 1,405 participants with all stages of CKD confirmed, with high certainty, that an average reduction in salt intake by 4.2 g per day reduces BP by 6.9/3.9 mmHg.^15^ Additionally, dietary salt reduction enhances the BP lowering, antiproteinuric and kidney protective efficacy of angiotensin receptor blockers.^15^, ^16^, ^17^ People with CKD should therefore be advised to limit dietary salt (sodium chloride) intake to <5 g per day.^18^

Adoption of modified Dietary Approaches to Stop Hypertension (DASH) or a ‘Mediterranean’ diet reduces BP, improves vascular function and metabolic health in the general population.^19^ A small subset (13%) of the original DASH-Sodium cohort had CKD (eGFR <60 mL/min/1.73 m^2^ or presence of albuminuria) and their BP response to the dietary intervention was not modified by the presence of CKD.^20^ A DASH diet did not adversely affect serum potassium in a small, short-term study.^21^

Randomised controlled trials and meta-analyses show that structured exercise (aerobic or combined aerobic–resistance training) reduces systolic and diastolic BP in CKD by approximately 4–6 mmHg and diastolic BP by 2–3 mmHg when performed over 12–26 weeks, with effects additive to antihypertensive medication.^22^, ^23^ However, studies are generally small and heterogeneous, and long-term (>12 months) BP‐lowering effects are less consistent. KDIGO guidelines recommend that people with CKD undertake moderate-intensity physical activity for a cumulative duration of at least 150 min per week.^18^

Weight management, limited alcohol consumption, smoking cessation and adequate sleep further support BP control. Multidisciplinary support and renal dietetic input are crucial to sustain these behavioural changes.

## Current pharmacological therapy

**Fig. 1:**
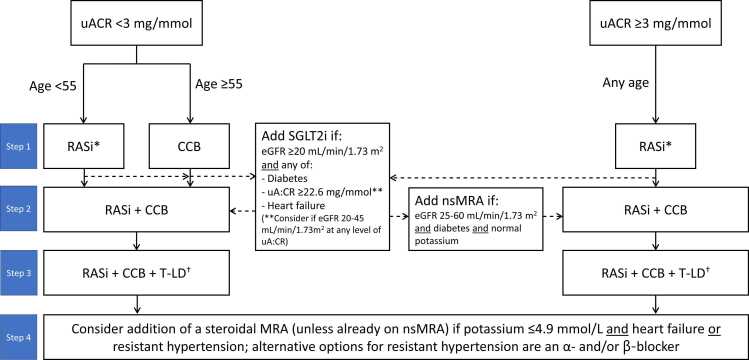
Algorithm for recommended combinations of antihypertensives and other nephroprotective medications when managing hypertension in CKD. *RASi dose should be increased to maximum tolerated dose; RASi should be continued even when eGFR falls below 30 mL/min/1.73 m^2^. ^†^Chlorthalidone in particular effectively lowers BP down to an eGFR of 15 mL/min/1.73 m^2^. RASi, renin–angiotensin system inhibitor; CCB, calcium channel blocker; TL-D, thiazide-like diuretic.

International (KDIGO)^18^, ^7^ and UK (NICE CG 136)^9^ guidelines recommend broadly similar approaches to pharmacological management of BP in CKD, with choice of agent being principally determined by presence or absence of diabetes, urine albumin:creatinine ratio (uACR) and eGFR. A suggested algorithm reflecting these various recommendations is presented in Fig. 1.

Sodium–glucose co-transporter inhibitors (SGLT2i) and non-steroidal mineralocorticoid receptor antagonists (nsMRAs) result in modest, but nonetheless clinically meaningful, reductions in office systolic BP (5.1 mmHg for SGLT2i^24^ and 2.1 mmHg for nsMRAs^25^) when used individually. Recently published data confirm a synergistic BP-lowering effect (SBP reduced by 7.4 mmHg from baseline) following simultaneous commencement of an SGT2i and nsMRA in type 2 diabetes, an approach confirmed as safe in the CONFIDENCE trial.^26^ Although not licensed in CKD *per se* (at least in the UK) and not approved for use by NICE, GLP-1 agonists also have modest effects on BP (placebo-corrected BP change of −2.2/+0.8 mmHg) in people with diabetic nephropathy.^27^

Other important and recently published clinical trials have confirmed the importance of continuation of renin–angiotensin system inhibitors (RASi) in those with eGFR <30 mL/min/1.73 m^2^ ^28^ and the efficacy of the thiazide-like diuretic chlorthalidone in those with eGFR 15–30 mL/min/1.73 m^2^ (−10.5 mmHg placebo-corrected change in ambulatory systolic BP).^29^

Hyperkalaemia frequently complicates attempts to achieve BP control, especially in those with diabetes or eGFR <30 mL/min/1.73 m^2^. Strategies to avoid cessation of nephroprotective RASi should include low potassium dietary advice, use of thiazide-like diuretics instead of steroidal MRAs when treating resistant hypertension and novel oral potassium binders such as sodium zirconium cyclosilicate.^30^

## Novel and emerging therapies

Hypertension in CKD is frequently resistant to conventional therapy, reflecting persistent activation of the RAS and aldosterone systems, sympathetic overactivity, sodium retention and endothelial dysfunction. Several novel therapeutic strategies are emerging to address these mechanisms.

Finerenone, a nsMRA, is established as an adjunctive treatment in CKD stage 3 and 4 with type 2 diabetes and is currently in a phase 3 clinical trial (FIND-CKD) in people with non-diabetic CKD.^31^ An alternative approach using aldosterone synthase inhibitors, including baxdrostat and lorundrostat, may represent a further important advance. These agents selectively inhibit CYP11B2, reducing aldosterone production without affecting cortisol synthesis. Phase 3 trials in uncontrolled and resistant hypertension have demonstrated substantial BP reductions when added to background therapy, with early evidence of albuminuria reduction.^32^, ^33^ Their role in CKD is particularly attractive given the central role of aldosterone in sodium retention, inflammation and fibrosis, although long-term renal outcome data for baxdrostat and vicadrostat are awaited from the PACIFIC and EASi-Kidney trials respectively.^34^, ^35^

Targeting the endothelin pathway is another novel approach. Aprocitentan, a dual endothelin-A/B receptor antagonist, significantly reduced BP in patients with resistant hypertension, including those with CKD, and was associated with reductions in albuminuria.^36^

Finally, renal denervation has re-emerged as a potential option for carefully selected patients with CKD and resistant hypertension, with recent sham-controlled trials and registries demonstrating sustained BP reductions, although data in advanced CKD remain limited and further study will be required in this patient cohort.^37^

## CRediT authorship contribution statement

**Swapnil Hiremath:** Writing – review & editing, Writing – original draft. **Tim Doulton:** Writing – review & editing, Writing – original draft. **Pauline A. Swift:** Writing – review & editing, Writing – original draft.

## Funding

This article did not receive any specific grant from funding agencies in the public, commercial or not-for-profit sectors.

## Declaration of competing interest

The authors declare the following financial interests/personal relationships which may be considered as potential competing interests: Dr Tim Doulton reports a relationship with AstraZeneca UK Limited that includes: speaking and lecture fees. Dr Tim Doulton reports a relationship with Boehringer Ingelheim Ltd that includes: consulting or advisory and speaking and lecture fees. Dr Tim Doulton reports a relationship with ReCor Medical Inc that includes: speaking and lecture fees. Dr Pauline A Swift reports a relationship with Vifor Pharma UK Ltd that includes: speaking and lecture fees and travel reimbursement. Dr Pauline A Swift reports a relationship with AstraZeneca UK Limited that includes: consulting or advisory, speaking and lecture fees, and travel reimbursement. The other authors declare that they have no known competing financial interests or personal relationships that could have appeared to influence the work reported in this paper.
